# Screening for HIV-Associated Tuberculosis and Rifampicin Resistance before Antiretroviral Therapy Using the Xpert MTB/RIF Assay: A Prospective Study

**DOI:** 10.1371/journal.pmed.1001067

**Published:** 2011-07-26

**Authors:** Stephen D. Lawn, Sophie V. Brooks, Katharina Kranzer, Mark P. Nicol, Andrew Whitelaw, Monica Vogt, Linda-Gail Bekker, Robin Wood

**Affiliations:** 1 The Desmond Tutu HIV Centre, Institute for Infectious Disease and Molecular Medicine, Faculty of Health Sciences, University of Cape Town, Cape Town, South Africa; 2 Clinical Research Unit, Department of Infectious and Tropical Diseases, London School of Hygiene and Tropical Medicine, London, United Kingdom; 3 Division of Medical Microbiology, Faculty of Health Sciences, University of Cape Town, Cape Town, South Africa; 4 National Health Laboratory Service, Groote Schuur Hospital, Cape Town, South Africa; 5 Department of Science and Technology/National Research Foundation Centre of Excellence in Epidemiological Modelling and Analysis, University of Stellenbosch, Cape Town, South Africa; McGill University, Canada

## Abstract

**Background:**

The World Health Organization has endorsed the Xpert MTB/RIF assay for investigation of patients suspected of having tuberculosis (TB). However, its utility for routine TB screening and detection of rifampicin resistance among HIV-infected patients with advanced immunodeficiency enrolling in antiretroviral therapy (ART) services is unknown.

**Methods and Findings:**

Consecutive adult HIV-infected patients with no current TB diagnosis enrolling in an ART clinic in a South African township were recruited regardless of symptoms. They were clinically characterised and invited to provide two sputum samples at a single visit. The accuracy of the Xpert MTB/RIF assay for diagnosing TB and drug resistance was assessed in comparison with other tests, including fluorescence smear microscopy and automated liquid culture (gold standard) and drug susceptibility testing. Of 515 patients enrolled, 468 patients (median CD4 cell count, 171 cells/µl; interquartile range, 102–236) produced at least one sputum sample, yielding complete sets of results from 839 samples. *Mycobacterium tuberculosis* was cultured from 81 patients (TB prevalence, 17.3%). The overall sensitivity of the Xpert MTB/RIF assay for culture-positive TB was 73.3% (specificity, 99.2%) compared to 28.0% (specificity, 100%) using smear microscopy. All smear-positive, culture-positive disease was detected by Xpert MTB/RIF from a single sample (sensitivity, 100%), whereas the sensitivity for smear-negative, culture-positive TB was 43.4% from one sputum sample and 62.3% from two samples. Xpert correctly identified rifampicin resistance in all four cases of multidrug-resistant TB but incorrectly identified resistance in three other patients whose disease was confirmed to be drug sensitive by gene sequencing (specificity, 94.1%; positive predictive value, 57%).

**Conclusions:**

In this population of individuals at high risk of TB, intensive screening using the Xpert MTB/RIF assay increased case detection by 45% compared with smear microscopy, strongly supporting replacement of microscopy for this indication. However, despite the ability of the assay to rapidly detect rifampicin-resistant disease, the specificity for drug-resistant TB was sub-optimal.

*Please see later in the article for the Editors' Summary*

## Abstract 2

In a prospective study, Stephen Lawn and colleagues find that pre-ART screening with Xpert MTB/RIF increased tuberculosis case detection by 45% compared to smear microscopy in HIV-positive patients at high risk of TB risk. AE competing interests must also pull through to the proof. “The Academic Editor, Madhukar Pai, declares that he consults for the Bill & Melinda Gates Foundation (BMGF). The BMGF supported FIND which was involved in the development of the Xpert MTB/RIF assay. He also co-chairs the Stop TB Partnership's New Diagnostics Working Group that was involved in the WHO endorsement of the Xpert assay.” Linked: Scott pmed.1001061; Evans pmed.1001064; Dowdy pmed.1001063

## Editors' Summary

**Background:**

Tuberculosis (TB)—a contagious bacterial infection that mainly affects the lungs—is a leading cause of illness and death among people who are infected with HIV, the virus that causes AIDS by destroying the immune system, which leaves infected individuals susceptible to other infections. TB is caused by *Mycobacterium tuberculosis*, which is spread in airborne droplets when people with the disease cough or sneeze. Its symptoms include a persistent cough, weight loss, and night sweats. Diagnostic tests for TB include chest X-rays, sputum smear analysis (microscopic examination of mucus coughed up from the lungs for *M. tuberculosis* bacilli), and mycobacterial liquid culture (the growth of *M. tuberculosis* from sputum and determination of its drug sensitivity). TB can be cured by taking several drugs daily for six months, although the recent emergence of multidrug-resistant TB (MDR-TB) is making the disease increasingly hard to treat.

**Why Was This Study Done?:**

TB is a major problem in clinics that provide antiretroviral therapy (ART) for HIV-positive people in resource-limited settings. Not only is it a major cause of sickness and mortality in those affected by it, but TB (especially MDR-TB) can also spread to other patients attending the same clinic for health services. Rapid diagnosis and appropriate treatment are very important to reduce these risks. Unfortunately, sputum smear analysis—the mainstay of TB diagnosis in resource-limited settings—only detects about a fifth of TB cases when used as a screening tool before initiating ART. Chest X-rays are costly and don't always detect TB, and liquid culture—the gold standard method for TB diagnosis—is costly, technically difficult, and slow. Consequently, the World Health Organization (WHO) recently endorsed a new test for the investigation of patients suspected of having TB, especially in regions where HIV infection and MDR-TB are common. Xpert MTB/RIF is an automated DNA test that detects *M. tuberculosis* and DNA differences that make the bacteria resistant to the drug rifampicin (an indicator of MDR-TB) within 2 hours. In this study, the researchers investigate whether Xpert MTB/RIF could be used as a routine screening test to increase TB detection among HIV-positive people initiating ART.

**What Did the Researchers Do and Find?:**

The researchers collected sputum from HIV-infected adults with no current TB diagnosis enrolling at an ART clinic in a South African township where HIV infection and TB are both common. They then compared the diagnostic accuracy of Xpert MTB/RIF (performed at a centralized laboratory) with that of several other tests, including liquid culture (the reference test). Nearly a fifth of the patients had culture-positive TB. Xpert MTB/RIF identified three-quarters of these patients (a sensitivity of 73.3%). By contrast, the sensitivity of smear microscopy was 28%. The new test's specificity (the proportion of patients with a negative Xpert MTB/RIF result among patients without TB) was 99.2%. That is, Xpert MTB/RIF had a low false-positive rate. Notably, Xpert MTB/RIF detected all cases of smear-positive, culture-positive TB but only 43.4% of smear-negative, culture-positive cases from a single sputum sample; it detected 62.3% of such cases when two sputum samples were analyzed. Finally, Xpert MTB/RIF correctly identified rifampicin resistance in all four patients who had MDR-TB but incorrectly identified resistance in three patients with drug-sensitive TB.

**What Do These Findings Mean?:**

In this population of HIV-positive patients with a high TB risk, pre-ART screening with Xpert MTB/RIF increased case detection by 45% compared to smear microscopy, a finding that needs confirming in other settings. Importantly, Xpert MTB/RIF reduced the delay in diagnosis of TB from more than 20 days to two days. This delay would be reduced further by doing the assay at ART clinics rather than at a centralized testing facility, but the diagnostic accuracy of point-of-care testing needs evaluating. Overall, these findings (and those of an accompanying article by Scott et al. that examines the performance of Xpert MTB/RIF in an area where HIV infection is common) support the replacement of smear microscopy with Xpert MTB/RIF for pre-ART TB screening (provided misdiagnosis of rifampicin resistance can be reduced). These findings also suggest that routine screening with Xpert MTB/RIF could reduce the risk of MDR-TB outbreaks in HIV care and treatment settings and improve outcomes for HIV-positive patients with MDR-TB who currently often die before a diagnosis of TB can be made.

**Additional Information:**

Please access these Web sites via the online version of this summary at http://dx.doi.org/10.1371/journal.pmed.1001056.

- This study is further discussed in a *PLoS Medicine* Perspective by Carlton Evans; a related *PLoS Medicine* Research Article by Scott et al. is also available
- WHO provides information (in several languages) on all aspects of tuberculosis, including general information on tuberculosis diagnostics and specific information on the Xpert MTB/RIF test; further information about WHO's endorsement of Xpert MTB/RIF is included in a recent Strategic and Technical Advisory Group for Tuberculosis report
- WHO also provides information about tuberculosis and HIV
- The US National Institute of Allergy and Infectious Diseases has detailed information on tuberculosis and HIV/AIDS
- The US Centers for Disease Control and Prevention also has information about tuberculosis, including information on the diagnosis of and on tuberculosis and HIV co-infection
- Information is available from Avert, an international AIDS charity, on many aspects of HIV/AIDS, including information on HIV-related tuberculosis (in English and Spanish)

## Introduction

Tuberculosis is a major challenge for antiretroviral therapy (ART) services in resource-limited settings where patients typically enrol with advanced immunodeficiency [1]. Many patients referred for ART have a current TB diagnosis, and an additional large burden of disease is detected during pre-treatment screening [2]–[4]. Tuberculosis in this population is a major cause of morbidity and mortality [1],[5]–[7] and presents a substantial hazard of nosocomial disease transmission to other patients and health care workers [8]. These risks are heightened when patients have multidrug-resistant TB (MDR-TB) [9]–[11]. To address these challenges, there is a critical need in such settings for rapid, effective screening for TB and detection of drug resistance [1],[12].

Screening for TB in this patient population is difficult, however [12]. The World Health Organization's (WHO) intensified case finding symptom screen has low specificity and misses approximately 10%–20% of cases [13],[14]. Sputum smear microscopy, the mainstay of TB diagnosis in resource-limited settings, detects as few as one in five cases when used as a screening tool pre-ART [4],[12],[15]. Chest radiography is costly and not widely available; interpretation is difficult, and up to one-third of culture-confirmed cases of pulmonary TB diagnosed during screening have a normal radiograph [12],[16]. Availability of culture-based diagnosis is also extremely limited in resource-limited settings because of high cost and technical complexity, and this approach often provides a diagnosis only after several weeks [15],[17]. These challenges are further compounded by the extremely limited laboratory capacity to detect drug resistance [18]. The threat posed by MDR-TB to efforts to control TB worldwide [19] requires urgent improvements in diagnostic capacity.

Following a large multi-country evaluation [20], the WHO, in December 2010, endorsed the roll-out of a novel rapid test for the investigation of patients suspected of having TB, especially in settings with a high prevalence of HIV-associated disease and/or MDR-TB [21]. The Xpert MTB/RIF assay (Cepheid) is a fully automated molecular assay in which real-time polymerase chain reaction technology is used to simultaneously detect *Mycobacterium tuberculosis* and rifampicin resistance mutations in the *rpoB* gene [22],[23]. The cartridge-based system dispenses with the need for prior sputum processing and requires minimal laboratory expertise, and results are available in less than 2 h, permitting a specific TB diagnosis and rapid detection of rifampicin resistance. Excellent performance characteristics were observed among symptomatic adults with suspected TB in a large multi-country evaluation [20]. These findings have been confirmed in a subsequent multi-country implementation study [24] and in several laboratory-based studies [25]–[29]. The assay has sensitivities of 98%–100% for smear-positive pulmonary TB, 57%–78% for smear-negative pulmonary TB, and 53%–81% for extrapulmonary TB when testing a variety of clinical samples [20],[24]–[29].

Further studies are needed to examine the performance of the assay in different clinical settings, including use as a routine screening test to increase TB case detection in HIV-infected patients. We evaluated the diagnostic accuracy of the Xpert MTB/RIF assay among consecutive patients with advanced immunodeficiency being screened for TB (regardless of symptoms) prior to starting ART in a South African township with a very high burden of TB.

## Methods

### Setting

The ART cohort was based in Gugulethu township, Cape Town, where the prevalence of HIV and the TB notification rate are both extremely high [5]. Several studies reporting the burden, diagnosis, and complications of TB in this cohort have previously been published [3],[5],[15],[16],[30],[31]. National TB programme guidelines recommend investigating symptomatic adults with suspected pulmonary TB using smear microscopy of two sputum samples; in suspected “retreatment TB” cases only, culture of one sputum sample may be requested in addition [32]. In accordance with the national ART programme guidelines, ART was provided for all patients with WHO stage 4 disease and/or blood CD4 cell counts <200 cells/µl and for pregnant women and patients with TB with CD4 cell counts <350 cells/µl. All patients gave written informed consent, and this study was approved by the human research ethics committees of the University of Cape Town and the London School of Hygiene and Tropical Medicine. This study conforms to the STARD initiative guidelines (http://www.stard-statement.org/) (Text S1) for reporting of studies of diagnostic accuracy.

### Patients and Investigations

Patients eligible for the study were ART-naive, were aged ≥18 y, and did not have a current TB diagnosis. Consecutive patients referred to the clinic were prospectively recruited and investigated at their first visit. Demographic details were recorded, and a standardised symptom-screening questionnaire was completed. Data collected included the WHO symptom screen (one or more of the following symptoms: current cough, fever, night sweats, or weight loss) [14]. Two sputum samples were requested from each patient; a spot specimen was first obtained, followed by a second sample that was induced using nebulised 3% hypertonic saline. If necessary, both specimens were induced. Chest radiographs were obtained on all patients except pregnant women and were evaluated by an experienced reader certified in the use of the chest radiograph reading and recording system [16],[33]. Radiographs were scored with regard to the presence of any radiographic abnormality consistent with a diagnosis of TB. Blood CD4 cell counts and plasma viral load were measured on all patients via the routine laboratory services. For patients subsequently found to have false-positive Xpert MTB/RIF assays, all clinical records at baseline and follow-up were reviewed to determine the clinical course and ascertain any further evidence to support or refute a TB diagnosis.

### Laboratory Procedures

Sputum specimens were processed using standardised protocols and quality assurance procedures by a centralised accredited laboratory that participated in the previous multi-country evaluation of the Xpert MTB/RIF assay [20]. Following decontamination with *N*-acetyl-L-cysteine and sodium hydroxide, centrifuged sputum deposits underwent microscopy, and following resuspension in phosphate buffer, equal volumes were tested by liquid culture and the Xpert MTB/RIF assay. The results of all tests were read by technologists blinded to the outcomes of the other assays. The length of time between sample collection and results being issued to the clinic was also recorded.

Smears stained with auramine O fluorescent stain were examined using fluorescence microscopy. Bacillary density was graded as scanty, 1+, 2+, and 3+, and all such smears were defined as “smear-positive”. Sputum pellets were also tested by trained technologists using the Xpert MTB/RIF assay as previously described [20],[22],[23]. Sample reagent (1.5 ml) was added to 0.5 ml of the resuspended sputum pellet and manually agitated twice at room temperature during a 15-min period. The inactivated material was then transferred to the test cartridge and inserted into the automated test platform, and the results were recorded.

Mycobacterial growth indicator tubes (MGITs, BD) were also inoculated and incubated for up to 6 wk. The time to automated growth detection was recorded. Culture isolates positive for acid-fast bacilli were identified as *M. tuberculosis* complex and assessed for genotypic resistance using the MTBDRplus assay (Hain Lifescience). Isolates also underwent phenotypic resistance testing for rifampicin and isoniazid by automated liquid MGIT culture (using the modified proportion method and standard protocols). For isolates found to have discrepant rifampicin susceptibility results using different assays, the *rpoB* region was sequenced using standard methods as previously described [20].

### Definitions and Analyses

Patients with *M. tuberculosis* cultured from one or more sputum samples were defined as cases of TB. Resistance to rifampicin and isoniazid was defined by phenotypic resistance typing using MGIT cultures wherever available; the remainder were defined by MTBDRplus assay testing of the culture isolate.

The study population was characterised using simple descriptive statistics, and patients with and without TB were compared using the Wilcoxon rank-sum test, *t*-test, chi-square test, or Fisher's exact test as appropriate. Disease prevalence was determined, and binomial regression analysis was used to identify factors associated with TB risk. Results of MGIT culture were compared with the results of the three other laboratory tests in a per-patient analysis. The sensitivity, specificity, positive predictive value (PPV), and negative predictive value (NPV) of the assays with 95% confidence intervals (95% CIs) were determined using Stata software. All statistical tests were two-sided at α = 0.05.

## Results

### Patients and TB Diagnoses

**Figure 1:**
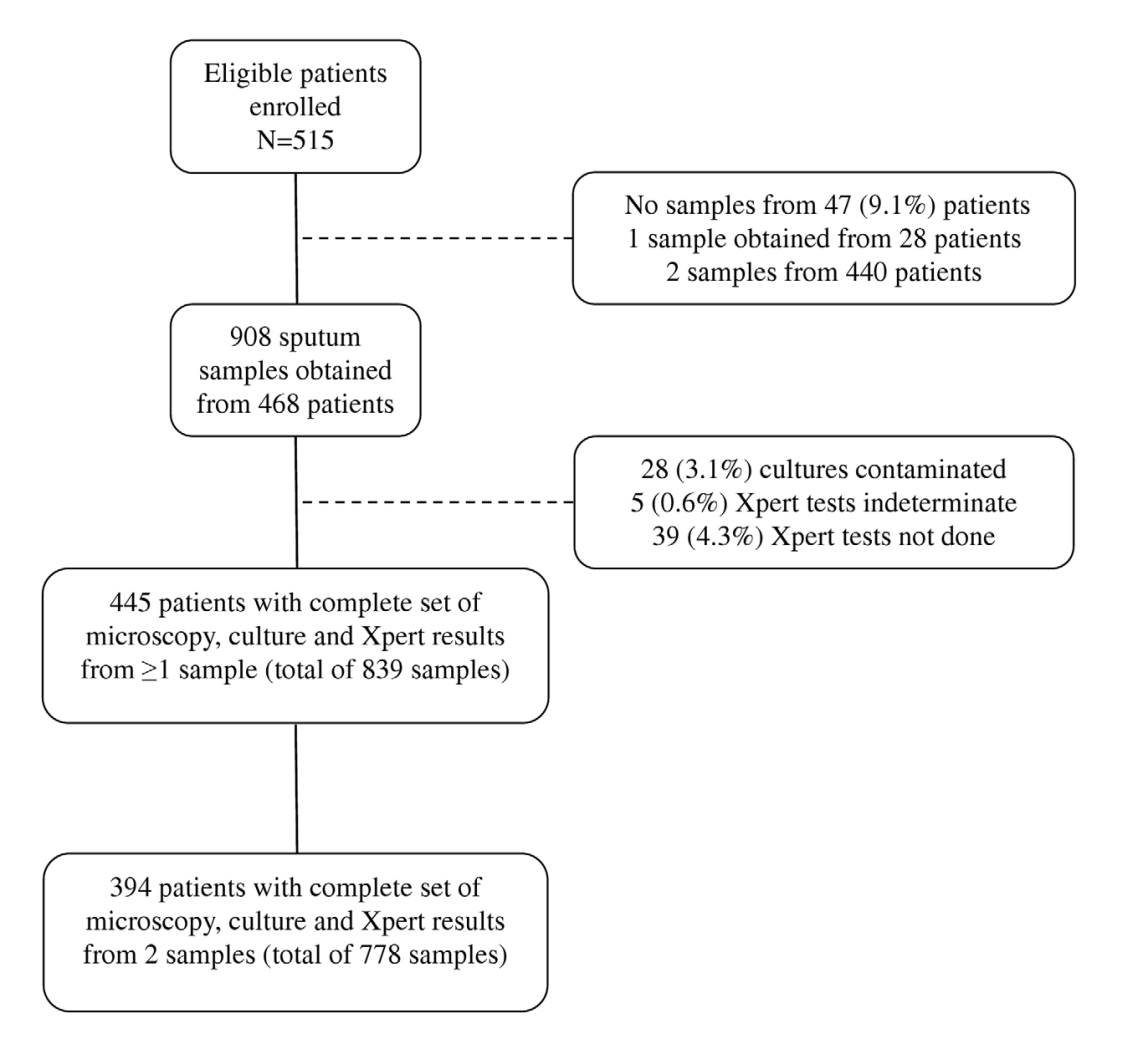
Flow diagram showing the numbers of patients enrolled, losses, numbers of sputum samples analysed, and numbers of results obtained.

Between 12 March 2010 and 9 February 2011, 515 of 517 consecutively invited patients agreed to participate in this study. A total of 908 samples were collected: two samples from 440 patients, one sample from 28 patients, and no sample from 47 patients (Figure 1). The vast majority of first sputum samples (89%) were obtained by spontaneous expectoration, and the remainder of first samples and all second samples were induced using hypertonic saline. From the 908 sputum samples obtained, 28 (3.1%) cultures were contaminated and were excluded (Figure 1). *M. tuberculosis* was cultured from a total of 137 samples, resulting in TB diagnoses in 81 patients. Of these, 67 (82.7%) were diagnosed from a first sputum sample, and an additional 14 (17.3%) cases from a second sample. Twenty five (30.9%) were sputum smear-positive cases for which the highest smear grades were scanty (*n* = 8), 1+ (*n* = 6), 2+ (*n* = 8), and 3+ (*n* = 3). The median time to positivity of MGIT cultures was 16 d (interquartile range [IQR], 11–20) overall (10 d for smear-positive disease; 18 d for smear-negative disease).

The characteristics of the patient population are shown in Table 1. Patients typically had advanced immunodeficiency (median CD4 cell count, 171 cells/µl), and 26.5% of patients had previously had TB. Compared to patients in whom no TB diagnosis was made, TB patients had a lower body mass index, lower CD4 cell counts, higher plasma viral loads, and more advanced WHO stage of disease at enrolment (Table 1). A positive WHO symptom screen was observed in 84% of TB patients (92% for smear-positive disease and 76% for smear-negative disease) compared to 67% of patients who were TB-free (*p*<0.01). However, TB patients were not significantly more likely to report chronic cough lasting ≥2 wk. Although radiological abnormalities were more common among patients with culture-confirmed TB, 28.9% of confirmed TB patients had a normal chest radiograph (Table 1). Conversely, 33.9% of patients who did not have TB had an abnormal radiograph.

**Table 1:** Characteristics of all patients (*n* = 468) for whom results of sputum cultures were available from one or more sputum samples.

Patient Characteristics^a^	All Patients (*n* = 468)	TB Diagnosed (*n* = 81)	No TB Diagnosed (*n* = 387)	*p*-Value^b^
**Age, median (IQR)**	33.6 (27.8–40.7)	32.1 (28.2–40.4)	33.6 (27.7–40.8)	0.70
**Female**	306 (65.4%)	54 (66.7%)	252 (65.1%)	0.79
**BMI, median (IQR)**	23.5 (20.9–27.2)	21.4 (19.1–25.9)	23.9 (21.1–27.6)	<0.001
**History of previous TB**	124 (26.5%)	16 (19.8%)	108 (27.9%)	0.13
**CD4 cell counts (cells/µl)**				
Median (IQR)	171 (102–236)	130.5 (51.5–206.6)	176 (112–243)	<0.001
CD4 <50	59 (12.6%)	20 (24.7%)	39 (10.1%)	0.006
CD4 50–99	55 (11.8%)	12 (14.8%)	43 (11.1%)	
CD4 100–149	90 (19.2%)	15 (18.5%)	75 (19.2%)	
CD4 150–199	85 (18.2%)	9 (11.1%)	76 (19.6%)	
CD4 ≥200	179 (38.3%)	25 (30.9%)	154 (39.9%)	
**Baseline viral load, median (IQR) (log_10_ copies/ml)**	4.5 (4.0–5.0)	4.8 (4.4–5.3)	4.5 (4.0–4.9)	<0.001
**WHO stage at enrolment**				
1 or 2	317 (67.7%)	45 (55.6%)	272 (70.3%)	0.009
3 or 4	151 (32.3%)	36 (44.4%)	115 (29.7%)	
**Positive WHO symptom screen**	328 (70.1%)	68 (84.0%)	260 (67.2%)	0.003
**Current cough ≥2 wk**	103 (22.0%)	22 (27.2%)	81 (20.9%)	0.22
**Radiological abnormality consistent with TB** c	170 (40.7%)	54 (71.1%)	116 (33.9%)	<0.001

a Data are number of patients (percent) unless otherwise indicated.

b Comparison of characteristics of patients with and without TB.

c Chest radiographs available for 418 patients.

### TB Prevalence and Risk Factors

The prevalence of culture-proven TB was 17.3% (95% CI, 13.9–20.7) among those from whom sputum could be obtained. The prevalence rates of sputum smear-positive and smear-negative disease were 5.3% and 12.0%, respectively. TB prevalence was strongly associated with baseline CD4 cell count. Prevalence rates among those with CD4 cell counts of <100 cells/µl, <200 cells/µl, and >200 cells/µl were 28.1% (95% CI, 19.7–36.4), 19.4% (95% CI, 14.7–24.0), and 13.8% (95% CI, 10.2–17.5), respectively. In binomial regression analysis (Table 2), risk of TB was independently associated with low CD4 cell count, low body mass index, high viral load, not previously having received TB treatment, and having a positive WHO symptom screen. However, risk of TB was not associated with chronic cough of ≥2 wk duration.

**Table 2:** Binomial regression analysis showing crude and adjusted risk ratios for the associations between risk of sputum culture-positive tuberculosis and patient characteristics.

Patient Characteristics	Crude Risk Ratio	95% CI	*p*-Value	Adjusted Risk Ratio	95% CI	*p*-Value
Age ≤30 y	1					
Age >30 y	0.90	0.61–1.34	0.62			
Male	1					
Female	1.06	0.70–1.61	0.79			
Body mass index 18–25 kg/m^2^	1			1		
Body mass index <18 kg/m^2^	2.32	1.44–3.75	0.001	2.94	1.30–6.63	0.009
Body mass index >25 kg/m^2^	0.68	0.42–1.09	0.109	0.70	0.39–1.27	0.243
No history of previous TB treatment	1			1		
History of previous TB treatment	0.68	0.41–1.13	0.14	0.50	0.26–0.96	0.036
CD4 ≥100 cells/µl	1			1		
CD4 <100 cells/µl	2.08	1.41–3.08	<0.001	2.01	1.17–3.45	0.011
Viral load <4.5 log copies/ml	1			1		
Viral load ≥4.5 log copies/ml	2.29	1.46–3.59	<0.001	2.12	1.22–3.69	0.008
No cough ≥2 wk	1					
Cough ≥2 wk	1.32	0.85–2.05	0.21			
Negative symptom screen	1			1		
Positive symptom screen	2.23	1.28–3.90	0.005	2.35	1.22–4.50	0.010

### Diagnostic Accuracy of Xpert MTB/RIF for Tuberculosis

Xpert MTB/RIF assay results were obtained from 864 samples. Tests were not done on 39 samples because of a laboratory clerical error that was not in any way related to sputum culture outcomes or patient status. Xpert MTB/RIF assays also gave indeterminate results for five (0.6%) samples, which were excluded from subsequent analyses. Of these five samples, three were culture-positive for *M. tuberculosis*. A second sputum sample was available for two of these, and Xpert MTB/RIF assays were positive in both. Non-tuberculous mycobacteria were cultured from a total of ten (1.1%) sputum samples from eight patients, but none of these samples was associated with a positive Xpert MTB/RIF test.

In analyses to determine the diagnostic accuracy of Xpert MTB/RIF for TB diagnosis, we used data from the 839 samples from 445 patients for which results of smear microscopy, MGIT culture, and Xpert MTB/RIF assays were all complete (Figure 1). Analyses were first done for all patients (*n* = 445) with results from either one or two samples. Just over one-quarter of TB cases (28.0%) were diagnosed using fluorescence microscopy, with 100% specificity (Table 3). In contrast, overall, 73.3% of culture-confirmed TB cases were diagnosed using the Xpert MTB/RIF assay, increasing case detection by 45.3% (95% CI, 32.7–57.9) compared to smear microscopy. The Xpert MTB/RIF assay detected all smear-positive cases (100% sensitivity) and just under two-thirds (63%) of smear-negative cases, with high specificity (Table 3). The PPV and NPV of the Xpert MTB/RIF assay were both 94.8% (Table 3).

**Table 3:** Per-patient analysis of data showing the sensitivity and specificity of the Xpert MTB/RIF assay for tuberculosis diagnosis compared to sputum smear microscopy, using sputum liquid culture as the gold standard.

Samples	Smear Microscopy				XPERT MTB/RIF Assay					
	Sensitivity	Specificity	PPV	NPV	Sensitivity			Specificity	PPV	NPV
	Culture-Positive Cases	Culture-Negative Patients			Culture-Positive Cases	Smear-Positive, Culture-Positive Cases	Smear-Negative, Culture-Positive Cases	Culture-Negative Patients		
**All patients with one or two sputum samples with complete results (** ***n*** ** = 445)**										
All samples	21/75 (28.0) 18.2–39.6	370/370 (100.0) 98.9–100.0	100 (83.9–100)	87.3 (83.7–90.3)	55/75 (73.3) 61.9–82.9	21/21 (100.0) 83.9–100.0	34/54 (63.0) 48.7–75.7	367/370 (99.2) 97.7–99.8	94.8 (85.6–98.9)	94.8 (92.1–96.8)
**Patients with results from paired sputum samples with complete results (** ***n*** ** = 394)**										
One sample	16/72 (22.2) 13.3–33.6	322/322 (100.0) 98.9–100.0	100 (79.4–100)	85.2 (81.2–88.6)	42/72 (58.3) 46.1–69.8	19/19 (100) 82.4–100	23/53 (43.4) 29.8–57.7	320/322 (99.4) 97.8–99.9	95.4 (84.5–99.4)	91.4 (88.0–94.1)
Two samples	19/72 (26.4) 16.7–38.1	322/322 (100.0) 98.8–100.0	100 (82.4–100)	85.9 (81.9–89.3)	52/72 (72.2) 60.4–82.1	19/19 (100) 82.4–100	33/53 (62.3) 47.9–75.2	319/322 (99.1) 97.3–99.8	94.5 (84.9–98.9)	94.1 (91.0–96.4)

Sensitivity and specificity data are number correct/total (percent) 95% CI.

A second analysis was restricted to patients with complete data from two sputum samples (778 samples from 394 patients). Analysis of this restricted set of data also showed that smear microscopy performed poorly, with one and two samples yielding just 22.2% and 26.4% of TB diagnoses, respectively, compared to 58.3% and 72.2% using the Xpert MTB/RIF assay (Table 3). The incremental yields of using Xpert on one and two sputum samples were 36.1% (95% CI, 23.6–48.6) and 45.8% (95% CI, 32.9–58.7), respectively. The Xpert MTB/RIF assay also identified all cases of smear-positive TB from a single sputum sample. Compared to the gold standard of MGIT cultures of two samples, the diagnostic yields of a single MGIT culture for all culture-positive, smear-positive, and smear-negative cases were 80.6% (95% CI, 69.5–88.9), 89.5% (95% CI, 66.9–98.7), and 77.4% (95% CI, 63.8–87.7), respectively.

The sensitivity of the Xpert MTB/RIF assay for smear-negative TB was substantially lower than for smear-positive disease and was dependent on the number of sputum samples, with sensitivities of 43.4% and 62.3% from one and two samples, respectively. In further analyses, factors associated with the sensitivity of the Xpert MTB/RIF assay for smear-negative disease were explored. Sensitivity was 100% for those with cough duration of >2 wk compared to 56.5% (95% CI, 41.6–71.4) among those with either no cough or cough of shorter duration (*p* = 0.018). Moreover, sensitivity was substantially greater in patients for whom the time to positivity of sputum samples was less than the median of 16 d (85.7%; 95% CI, 69.4–100) than in those with longer times to positivity (48.5%; 95% CI, 30.4–66.5) (*p* = 0.005). There was also a weak association between sensitivity and CD4 cell counts: sensitivity was 78.9% (95% CI, 58.8–99.1) in those with CD4 cell counts <100 cells/µl compared to 54.3% (95% CI, 36.9–71.6) in those with higher CD4 cell counts (*p* = 0.075). However, there was no association with radiographic abnormalities or with a positive WHO symptom screen.

There were three patients with apparent false-positive Xpert MTB-RIF assays, giving an assay specificity of over 99.0% in each of the different analyses (Table 3). Review of the study and clinical records of these patients revealed that two of these patients had overt pulmonary and systemic symptoms suggestive of TB, and both had chest radiographs revealing parenchymal consolidation and marked hilar and paratracheal lymphadenopathy highly suggestive of TB. One of these patients was reinvestigated during routine clinical follow-up and had two positive sputum smears (2+ and 3+). Both patients received standard treatment for TB and made excellent clinical responses. The third patient had symptoms and an abnormal chest radiograph but was lost to follow-up.

### Use of Xpert MTB/RIF in Screening Algorithms

To further explore the utility of the Xpert MTB/RIF assay, we considered clinical populations with a TB prevalence of 20%, 15%, 10%, or 5%. With an overall sensitivity of 73.3% and specificity of 99.2% (Table 3), the PPVs at these TB prevalence rates would be 95.8%, 94.2%, 91.0%, and 82.8%, respectively, and the NPVs would be 93.7%, 95.5%, 97.1%, and 98.6%, respectively.

We next considered the utility of incorporating the Xpert MTB/RIF assay into different screening algorithms, examining the use of smear microscopy, symptom screening, one Xpert assay, two Xpert assays (Xpert done on a second sample if the first was negative), and sequential smear microscopy and Xpert testing (Xpert tests done if smear microscopy was negative). This was simulated for a hypothetical cohort of 1,000 patients with a TB prevalence of 20%, 15%, 10%, or 5% and assuming that 30% of cases were smear-positive. Symptom frequencies and the sensitivity and specificity of the Xpert assay as reported above were used.

The yield of TB cases, the number of missed cases, and the number of Xpert tests done for each correct TB diagnosis were compared between these different screening strategies and clinical populations (Table 4). Compared to a base case scenario of smear microscopy of two sputum samples in patients with a positive WHO symptom screen, the sensitivity of algorithms incorporating the Xpert MTB/RIF assay was much greater and the corresponding number of missed diagnoses was far fewer. However, at a TB prevalence of 5%, the number of Xpert tests done per case diagnosed was high (Table 4). A strategy of sequential smear microscopy and then Xpert testing of smear-negative patients yielded the same number of diagnoses, but did not substantially reduce the number of Xpert tests per case diagnosed.

**Table 4:** Utility of the Xpert MTB/RIF assay for tuberculosis diagnosis when incorporated into different screening algorithms and when used in hypothetical patient cohorts with a tuberculosis prevalences of 20%, 15% 10%, or 5%.

Investigation Strategy	Sensitivity (Percent)^a^	Specificity (Percent)	TB Prevalence 20%			TB Prevalence 15%			TB Prevalence 10%			TB Prevalence 5%		
			Correct TB Diagnoses	Missed TB Cases	Xpert Tests per TB Diagnosis	Correct TB Diagnoses	Missed TB Cases	Xpert Tests per TB Diagnosis	Correct TB Diagnoses	Missed TB Cases	Xpert Tests per TB Diagnosis	Correct TB Diagnoses	Missed TB Cases	Xpert Tests per TB Diagnosis
**Base case screening algorithm**														
Symptom screen + smear ×2	27.6	100.0	55.2	144.8	0	41.4	108.6	0	27.6	72.4	0	13.8	36.2	0
**Using one Xpert test in algorithm**														
Symptom screen+Xpert ×1	50.5	99.6	101	99	6.9	75.7	74.3	9.1	50.5	49.5	13.5	25.2	24.8	26.9
Symptom screen+smear ×2+Xpert ×1	50.5	99.6	101	99	6.4	75.7	74.3	8.6	50.5	49.5	13.1	25.2	24.8	26.3
Xpert ×1 for all patients	60.1	99.4	120.2	79.8	8.3	90.2	59.8	11.1	60.1	39.9	16.6	30.1	19.9	33.2
Smear ×2+Xpert ×1 for all patients	60.1	99.4	120.2	79.8	7.8	90.2	59.8	10.6	60.1	39.9	16.1	30.1	19.9	32.7
**Using two Xpert tests in algorithm**														
Symptom screen+Xpert ×2	60.6	99.4	121.2	78.8	11.1	90.9	59.1	14.7	60.6	39.4	22.1	30.2	19.8	44.4
Xpert ×2 for all patients	73.4	99.1	146.8	53.2	13.2	110.1	39.9	17.8	73.4	26.6	26.8	36.7	13.3	54.1

a Sensitivity based on the assumption that 30% of cases are sputum smear-positive.

Use of symptom pre-screening limited the sensitivity of TB detection. In populations with high TB prevalence, Xpert testing of all patients regardless of symptoms increased sensitivity without substantially increasing the number of Xpert tests done per TB case diagnosed (Table 4). Compared to the strategy of doing an Xpert assay on one sputum sample from patients with a positive symptom screen, a strategy of doing two Xpert tests on all patients was associated with 22.9% higher sensitivity for TB and the fewest missed cases. Although the latter strategy would require a large absolute number of tests, at a TB prevalence of 20%, one extra TB case would be diagnosed for every additional 6.3 tests done.

### Detection of Rifampicin Resistance

Among 81 cases of TB diagnosed, four cases had isolates resistant to rifampicin because of MDR-TB (prevalence, 4.9%; 95% CI, 1.4–12.2). Among the 445 patients (839 samples) with results of culture, drug susceptibility testing, and Xpert MTB/RIF assays all available, there were 84 isolates from 55 patients (including all four cases of MDR-TB) in which rifampicin susceptibility could be compared. Rifampicin resistance was correctly identified in all four cases of MDR-TB by the Xpert MTB/RIF assay (100% sensitivity) (Table 5). However, the Xpert MTB/RIF assay also reported rifampicin resistance in three samples from three further patients in which the isolates were reported as rifampicin susceptible using comparator assays (Table 5). A paired sputum sample was available from two of these patients and rifampicin-susceptible *M. tuberculosis* was reported by Xpert MTB/RIF assay in both. To resolve these discrepancies, the *rpo*B regions of all five isolates from these three patients were sequenced. All were found to be wild-type, confirming absence of genotypic rifampicin resistance and indicating that the three Xpert MTB/RIF assay results were false positives. All remaining patients with susceptible isolates were correctly identified as such by the assay. Thus, in a per-patient analysis, the PPV of the Xpert MTB/RIF assay for detecting rifampicin resistance was 4/7 (57%) and the specificity was 48/51 (94.1%; 95% CI, 84.8–98.8).

**Table 5:** Comparison of results regarding drug susceptibility testing for rifampicin among paired samples from patients (*n* = 6) in whom rifampicin resistance was detected using one or more assays.

Patient Number	Sputum Smear	Xpert MTB/RIF	MTBDRplus on Sputum	MTBDRplus on Culture Isolate	MGIT Phenotypic DST	*rpoB* Gene Sequencing	Final Rifampicin Susceptibility	Overall Susceptibility Pattern
**Concordant susceptibility results**								
#020	NEG/NEG	−/R	−/−	−/R	−/R	−	Resistant	MDR-TB
#099	POS/POS	R/R	−/R	R/R	−/−	−	Resistant	MDR-TB
#208	NEG/NEG	R/−	−/−	R/R	R/R	−	Resistant	MDR-TB
#292	NEG/POS	R/R	R/−	R/R	R/−	−	Resistant	MDR-TB
**Discordant susceptibility results**								
#039	NEG/NEG	R/S	S/−	S/S	S/S	WT/WT	Susceptible	Pan-susceptible
#157	POS/POS	R/S	S/S	S/S	S/S	WT/WT	Susceptible	Pan-susceptible
#322	POS	R	−	S	S	WT/WT	Susceptible	Pan-susceptible

DST, drug susceptibility testing; NEG, smear-negative; POS, smear-positive; R, resistant; S, susceptible; WT, genotypically wild-type.

### Time to Diagnosis

The median delays between sputum collection and results being available to the clinic for smear microscopy and Xpert MTB/RIF assays and positive liquid cultures were 3 d (IQR, 2–5) and 4 d (IQR, 3–6), respectively. The median delays for culture results were 12 d (IQR, 10–14) and 20 d (IQR, 17–27) for smear-positive and smear-negative disease, respectively. Cultures were incubated for 42 d before being declared negative for *M. tuberculosis*, with a median time to reporting of 43 d (IQR, 43–45). For the patients with confirmed MDR-TB (*n* = 4), the mean time to TB diagnosis and detection of rifampicin resistance was 2 d using Xpert MTB/RIF assay, 21 d using the MTBDRplus assay on a positive culture isolate, and 40 d using phenotypic drug susceptibility testing in liquid culture.

## Discussion

A high prevalence (17.3%) of culture-proven pulmonary TB was diagnosed in this patient population, but conventional diagnostic tools widely used in resource-limited settings performed poorly. Smear microscopy detected just 28% of cases, and chest radiology was of low discriminatory value. Even using automated liquid culture as the diagnostic gold standard, diagnosis was slow, with a median delay of almost 3 wk among those with smear-negative disease. In contrast, the Xpert MTB/RIF assay was able to diagnose with extremely high specificity all cases of smear-positive TB and almost two-thirds of smear-negative cases and three-quarters of cases overall when testing two samples. Only 0.6% of test results were indeterminate. The assay also rapidly detected rifampicin resistance in all four cases of confirmed MDR-TB. However, false-positive rifampicin resistance results were also observed.

The TB prevalence and associated risk factors detected in this clinical setting were similar to those previously reported from this and another ART clinic in South Africa [3],[4],[15]. Almost 30% of patients with CD4 cell counts <100 cells/µl had culture-proven TB, and rapid diagnosis is needed since such patients have high mortality risk [5],[34]. Only one-quarter of all TB patients reported a cough lasting ≥2 wk—a symptom screen widely used for many years to define suspected TB cases. Use of the new WHO symptom screening tool [13],[14] had higher sensitivity but still would have missed 13 of the 81 TB diagnoses made in this study, suggesting the need for routine microbiological screening of all patients in this setting.

We evaluated the utility of the Xpert MTB/RIF assay as a screening tool in consecutive HIV-infected adult patients enrolling for ART, excluding those who already had a TB diagnosis (approximately one-third of referrals to this cohort [35]). Since patients were screened regardless of the presence or absence of symptoms, our study is likely to have diagnosed TB cases at an earlier stage in the disease course than studies in which symptomatic patients were tested. In contrast, the previous Foundation for Innovative New Diagnostics multi-country evaluation [20] enrolled only patients with overt TB symptoms; all had a chronic cough of at least 2 wk duration and were able to produce three 1.5-ml sputum specimens. Early disease in our study would tend to be associated with lower bacillary numbers in sputum samples, as indicated by the observations that almost 70% of cases were sputum smear-negative and the prolonged median time to positivity of liquid cultures. This patient population therefore represents a major challenge for any diagnostic assay [17]. The limits of detection of the Xpert MTB/RIF assay (95% sensitivity) defined by in vitro experiments is 131 bacilli/ml of sputum, which approaches than that of liquid culture, which falls within the range 10–100 bacilli/ml [17],[23]. In contrast, smear microscopy is able to detect only samples with more than approximately 10,000 organisms per millilitre [17],[23].

Testing a single sputum sample using Xpert MTB/RIF allowed diagnosis of all smear-positive cases regardless of smear grade; these cases pose the greatest infectious hazard within the community and health care settings. As anticipated [17], the sensitivity for smear-negative disease was lower than that reported in the previous multi-country evaluation [20] (43.3% versus 72.5% using one sputum sample; 63.3% versus 85.1% using two samples). Presence of cough of ≥2 wk was associated with much higher sensitivity for smear-negative TB, as was shorter time to culture positivity. The latter observation suggests that sensitivity was likely to have been limited by very low numbers of bacilli in sputum samples.

Three patients had false-positive TB diagnoses using Xpert MTB/RIF compared to the predefined laboratory gold standard of liquid culture. However, the clinical and radiological features in these cases were highly suggestive of TB; one was confirmed as having smear-positive TB on reinvestigation, two exhibited excellent responses to TB treatment, and the third patient was lost to follow-up. These follow-up data suggest that some or all of these false-positive Xpert MTB/RIF assays may actually have been correct. The proportion of cultures lost to contamination was very low (3.1%), highlighting possible over-decontamination in the laboratory and loss of sensitivity in the culture gold standard. If this was the case, the PPV of the assay would be higher, which would increase assay utility, especially in clinical populations with lower disease prevalence. Few Xpert MTB/RIF assays were indeterminate, but the observation that three out of five of these were in culture-positive cases suggests that indeterminate results should be followed up by a repeat test.

Despite only moderate sensitivity for smear-negative disease, Xpert MTB/RIF nevertheless increased overall case detection by 36% when testing one sample and by 45% when testing two samples, compared to smear microscopy. Used for baseline screening evaluation of patients enrolling in this ART service, Xpert MTB/RIF testing of a single sputum sample would detect TB in approximately 10% of the cohort, and testing two samples would detect TB in 12.5%. Thus, the assay would detect approximately one TB case for every eight patients screened, compared to one in 18 patients screened using sputum microscopy.

We explored the potential impact of incorporating the assay in several screening algorithms applied to clinical populations with a range of TB prevalence rates. The likely benefits (increased TB yield) and assay costs (tests done per case diagnosed) were highly dependent on TB prevalence, and at a prevalence rate of 5%, the number of tests done per case diagnosed was high (4-fold higher than for a population with a prevalence of 20%). A strategy of screening with sputum microscopy and then testing smear-negative samples with Xpert MTB/RIF assay would result in minimal savings with regard to the number of Xpert tests done but would also result in failure to diagnose MDR-TB in highly infectious smear-positive cases. Symptom pre-screening restricted sensitivity and, at higher TB prevalence rates, did not substantially reduce the number of Xpert MTB/RIF tests done to identify one case of TB when compared to a strategy of testing all patients regardless of symptoms. Screening two samples with Xpert MTB/RIF would substantially increase the absolute number of tests done, but at high TB prevalence rates the high incremental yield may justify this approach. The number of Xpert MTB/RIF assays done might logically be stratified by CD4 cell count since this is a strong predictor of TB prevalence. For example, in high-burden settings such as South Africa, two tests might be done for those with CD4 cell count <200 cells/µl and just one test for those with higher counts. These strategies need to be evaluated by detailed cost-effectiveness analyses that take into account not simply the costs of testing but also the downstream impact on clinical outcomes and associated costs.

Since the Xpert MTB/RIF instrument was based in a centralised laboratory service, with results reported via the routine laboratory system, the median time to diagnosis was similar to that of smear microscopy (4 d versus 3 d, respectively). The time to diagnosis of smear-negative disease, however, was shortened by a median of 2 wk compared to culture. Time to diagnosis and treatment would potentially be further shortened by location of the instrument in the ART clinic [24]. The assay also has the potential to shorten the time to exclude a diagnosis of TB; this normally takes 6 wk or more via negative cultures and may lead to inappropriate delays in ART initiation. In view of the high NPV of the Xpert MTB/RIF assay in this cohort (94.8%), a negative result at baseline evaluation could provide a useful indication of a low probability of TB, increasing clinical confidence to start ART without undue delay. In cohorts with a lower prevalence of TB, the NPV would be higher, further increasing its utility in this regard.

HIV-associated MDR-TB carries a high mortality risk, and nosocomial outbreaks in HIV care and treatment centres pose a grave threat to patients accessing these services [9],[10],[36]. Many patients with HIV-associated MDR-TB die before a diagnosis can be made [9],[36]. In this study, the Xpert MTB/RIF assay identified four patients with rifampicin-resistant isolates who had MDR-TB, greatly reducing the mean time to detection (2 d) compared to using conventional culture-based susceptibility testing (40 d) or using line probe assays on culture isolates (20 d). By accelerating diagnosis, the Xpert MTB/RIF assay has the potential to substantially reduce the risks of nosocomial transmission of MDR-TB and improve the prognosis of affected individuals.

The Xpert MTB/RIF assay reported three false-positive rifampicin resistance results. The finding of discordant rifampicin susceptibility results from paired samples using the Xpert MTB/RIF assay suggests that specificity might be increased by requiring confirmation of resistance in more than one sample. While such false positives were not found in the initial multi-country evaluation [20], another ongoing field study sponsored by the Foundation for Innovative New Diagnostics has also detected cases, leading the manufacturer to modify the instrument software and cartridge specifications [24],[37]. With WHO approval of roll-out of this assay in December 2010, confirmation of successful reconfiguration of the test platform is urgently required.

Strengths of the study include the use of a quality-assured laboratory that participated in the previous multi-country evaluation [20]. Whereas all previously published studies have evaluated use of the assay among individuals with suspected TB [20],[24]–[29], this study evaluated the assay as a screening tool in unselected consecutive patients regardless of symptoms in a high-burden setting. The TB status of all patients was clearly defined based on a rigorous laboratory gold standard. Weaknesses include the fact that a small number of tests were not done because of a laboratory clerical error and that there were few cases of MDR-TB. While a similar burden of disease has been reported from an ART clinic elsewhere in South Africa [4], the prevalence of TB may differ in other countries, and we therefore explored utility at a range of prevalence rates. The impact of the sputum concentration procedure and of dividing the sputum pellet between three assays rather than testing unprocessed sputum was not investigated in this study, but these methods were not found to impact assay sensitivity in a previous large-scale multi-country evaluation [20]. The usefulness of the assay as a point-of-care test was not evaluated. Further studies are needed to assess the impact of Xpert MTB/RIF screening on subsequent patient outcomes, the operational feasibility of using the assay within the clinic, and cost-effectiveness.

In conclusion, when used as a routine screening test among patients with advanced immunodeficiency and high TB risk, rapid screening using the Xpert MTB/RIF assay substantially increased case detection, supporting replacement of microscopy as the initial diagnostic tool. The assay also greatly decreased the time to diagnosis of MDR-TB. Use of Xpert MTB/RIF as a screening tool might effectively reduce the risk of nosocomial MDR-TB outbreaks in HIV care and treatment settings and improve the prognosis of affected patients. However, the specificity of the assay for detecting rifampicin resistance needs to be improved to prevent overdiagnosis of rifampicin-resistant disease.

## Supporting Information

Text S1 **STARD checklist.**
(PDF)

## Abbreviations

ART: antiretroviral therapy
CI: confidence interval
IQR: interquartile range
MDR-TB: multidrug-resistant tuberculosis
MGIT: mycobacterial growth indicator tube
NPV: negative predictive value
PPV: positive predictive value
TB: tuberculosis
WHO: World Health Organization
